# The Potential Role of Cathepsin K in Non-Small Cell Lung Cancer

**DOI:** 10.3390/molecules25184136

**Published:** 2020-09-10

**Authors:** Hui Yang, Jasmine Heyer, Hui Zhao, Shengxian Liang, Rui Guo, Li Zhong

**Affiliations:** 1 College of Life Sciences, Institute of Life Science and Green Development, Hebei University, Baoding 071000, China; huiyanglucky@163.com (H.Y.); huizhaols@yahoo.com (H.Z.); liangshengxiansr@163.com (S.L.); 2 College of Osteopathic Medicine of the Pacific, Western University of Health Sciences, Pomona, CA 91766, USA; jheyer44@gmail.com

**Keywords:** NSCLC, Cathepsin K, cell proliferation, cell migration, cell invasion, mTOR

## Abstract

(1) Background: Cathepsin K has been found overexpressed in several malignant tumors. However, there is little information regarding the involvement of Cathepsin K in non-small cell lung cancer (NSCLC). (2) Methods: Cathepsin K expression was tested in human NSCLC cell lines A549 and human embryo lung fibroblast MRC-5 cells using Western blot and immunofluorescence assay. Cathepsin K was transiently overexpressed or knocked down using transfection with a recombinant plasmid and siRNA, respectively, to test the effects on cell proliferation, migration, invasion, and on the mammalian target of rapamycin (mTOR) signaling pathway. (3) Results: Expression of Cathepsin K was increased significantly in A549 cells and diffused within the cytoplasm compared to the MRC-5 cells used as control. Cathepsin K overexpression promoted the proliferation, migration, and invasion of A549 cells, accompanied by mTOR activation. Cathepsin K knockdown reversed the above malignant behavior and inhibited the mTOR signaling activation, suggesting that Cathepsin K may promote the progression of NSCLC by activating the mTOR signaling pathway. (4) Conclusion: Cathepsin K may potentially represent a viable drug target for NSCLC treatment.

## 1. Introduction

Non-small cell lung cancer (NSCLC) is the leading cause of cancer death among men and women worldwide, with an incidence rate of 1.3 million cases per year [1]. Since NSCLC remains asymptomatic during early stages, about 80% of patients are already in metastatic stages when they are diagnosed, and their 5-year survival rates are below 15% [2]. Despite recent progress in the development of novel medications and immunotherapies in treating NSCLC, the therapeutic efficacies still remain unsatisfactory [3]. Therefore, finding new therapeutic targets for the treatment of NSCLC has become a prioritized task [4].

Cathepsin K is a type of lysosomal cysteine protease which belongs to the papain-like cysteine peptidase family; other members include Cathepsin B, Cathepsin D and Cathepsin L etc. Physiologically, Cathepsin K functions in mediating cellular protein turnover, collagen degradation, and remodeling of the extracellular matrix, which plays an important role in pulmonary fibrosis [5,6,7,8]. Deficiency of Cathepsin K can lead to severe bone abnormalities, as it is the main peptidase involved in bone remodeling in osteoclasts [9,10]. In addition, increased expression and activity of Cathepsin K have been reported in patients diagnosed with breast cancer [11], bone cancer [12], prostate cancer [13], and many other types of epithelial-derived cell cancers [14,15,16]. Similar to the dysregulated Cathepsin B expression in the tumor microenvironment inducing tumor progression [17], Cathepsin K over-expression is associated with cancer metastatic disease, indicating its potential diagnostic and prognostic value. Distinct expression patterns of Cathepsin K have been identified in lung cancer cells and stromal cells, which provide further supporting evidence for this protease’s significant prognostic value [18]. However, the specific role and mechanism of Cathepsin K in NSCLC is still unknown.

In this study, we revealed the effect of Cathepsin K expression on NSCLC cells in terms of cell proliferation, migration, and invasion in vitro. In order to understand the mechanisms, we also investigated the mammalian target of rapamycin (mTOR) signaling pathway. The mTOR signaling pathway plays an important role in maintaining cell growth, proliferation, motility, and survival [19]. Upregulation of the mTOR pathway has also been reported in a large number of NSCLC tumors, with increased p-mTOR expression in up to 90% of patients with adenocarcinoma, 60% of patients with large cell carcinoma, and 40% of patients with squamous cell carcinoma [20,21,22]. mTOR activation may also be associated with poor prognosis in early NSCLC [23,24]. Thus, mTOR inhibitors have been widely studied and employed clinically in order to suppress tumor growth and sensitize cells to anticancer drugs. Previous studies have demonstrated that inhibition of Cathepsin K can significantly reduced the phosphorylation of mTOR at S2448 in Caki cells [25]. Therefore, Cathepsin K may mediate activation of the mTOR signaling pathway in NSCLC. Our findings indicate that Cathepsin K has the potential in developing as a therapeutic target for NSCLC.

## 2. Results

### 2.1. Cathepsin K Was Highly Expressed in A549 Cells and Diffused in the Cytoplasm

**Figure 1:**
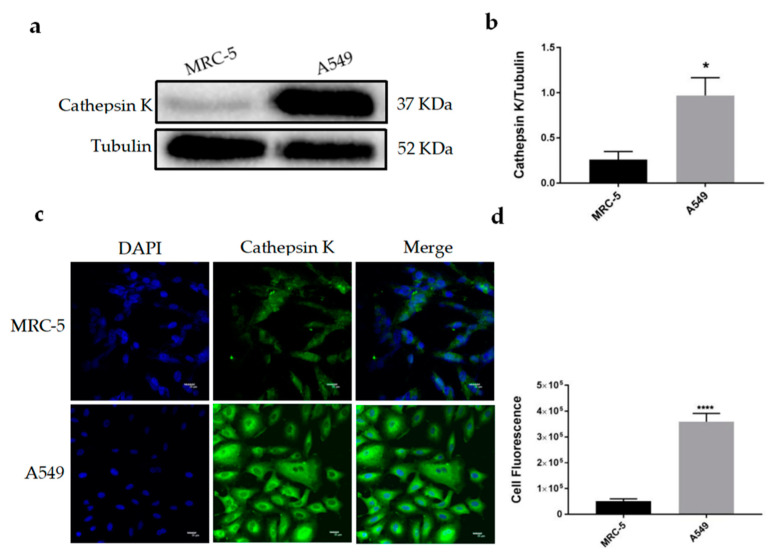
Detection of the expression and location of Cathepsin K in cells. (**a**) Western blot (WB) analysis for Cathepsin K expression in MRC-5, A549 cells. Representative gel blots of Cathepsin K and Tubulin using specific antibodies. (**b**) Cathepsin K/Tubulin; (**c**) Cathepsin K immunofluorescence (IF) staining in MRC-5 and A549 cells. (**d**) The cell fluorescence intensity was calculated using Image J software (mean ± SEM, *n* ≥ 3, * *p* ≤ 0.05, ** *p* ≤ 0.01, *** *p* ≤ 0.001).

Endogenous expression of Cathepsin K was detected in human embryonic lung fibroblasts MRC-5 and NSCLC cells A549 using a Western blot (WB) analysis and immunofluorescence (IF) assay. As shown in Figure 1a,b of WB results, compared to MRC-5 cells, the expression levels of Cathepsin K in A549 cells were significantly increased. An IF assay revealed that Cathepsin K was slightly expressed in MRC-5 cells, while Cathepsin K in A549 cells was largely expressed and diffused in the cytoplasm (Figure 1c), the cell fluorescence intensity was significantly increased (Figure 1d).

### 2.2. Cathepsin K Overexpression and Silence Models Were Successfully Reconstructed In Vitro

**Figure 2:**
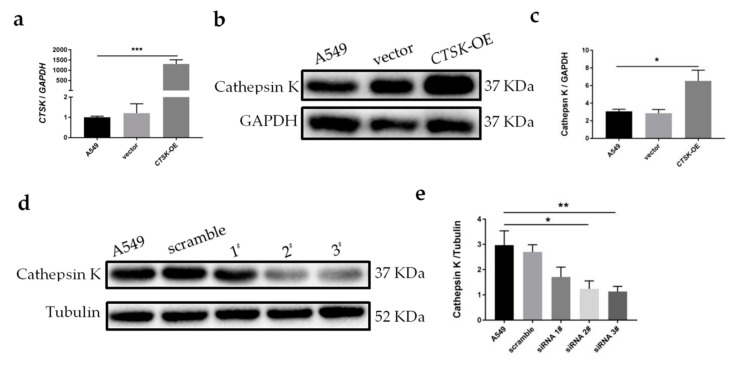
Construction of the Cathepsin K overexpression and knockdown models in vitro. (**a**) qRT-PCR analysis for *CTSK* expression in cells. *CTSK/GAPDH*; (**b**) WB analysis for Cathepsin K expression in cells. Representative gel blots of Cathepsin K and Tubulin using specific antibodies. (**c**) Cathepsin K/Tubulin; (**d**) WB analysis for Cathepsin K expression in cells. Representative gel blots of Cathepsin K and Tubulin using specific antibodies. (**e**) Cathepsin K/Tubulin (mean ± SEM, *n* ≥ 3, * *p* ≤ 0.05, ** *p* ≤ 0.01, *** *p* ≤ 0.001).

In order to observe the phenotypic variations mediated by Cathepsin K, it was transiently overexpressed or knocked down using transfection with a recombinant plasmid and siRNA into A549 cells respectively. As shown in Figure 2a, Cathepsin K mRNA expression levels were significantly increased in the Cathepsin K overexpression cells (*CTSK*-OE) compared to control A549 cells. The protein levels of Cathepsin K were also increased 1.5 times compared to A549 cells, and increased 1.6 times compared to the vector group (Figure 2b,c). Three Cathepsin K siRNA sequences were constructed for transfection, and the optimal sequence was selected according to its silencing efficiency. The results revealed that both siRNA 2# and siRNA 3# can effectively and markedly silence Cathepsin K expression (Figure 2d,e). The 3# sequence was selected to construct the Cathepsin K knock down (*CTSK*-KD) model.

### 2.3. CTSK-OE Promoted the Proliferation, Migration and Invasion and CTSK-KD Inhibited the Proliferation, Migration, and Invasion of A549 Cells

**Figure 3:**
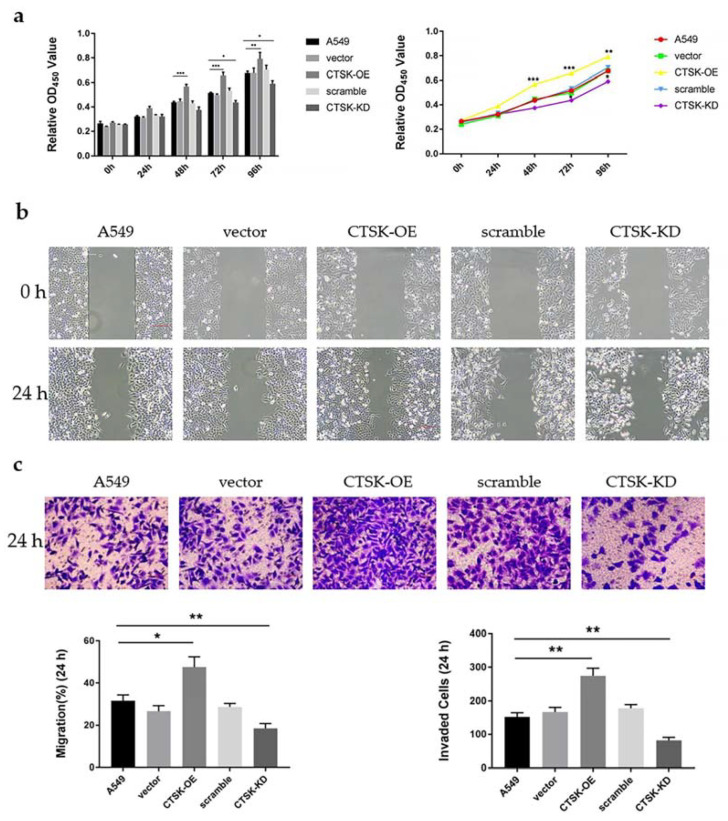
Detection of cell behavior in vitro. (**a**) Cell Counting Kit-8 (CCK-8) analysis for A549 cell proliferation. (**b**) Cell scratch repair analysis for A549 cell migration. (**c**) Invasion experiment analysis for A549 cell invasion (mean ± SEM, *n* ≥ 3, * *p* ≤ 0.05, ** *p* ≤ 0.01, *** *p* ≤ 0.001).

In order to understand whether *CTSK*-OE and *CTSK*-KD could have a significant effect on the proliferation, migration, and invasiveness of A549 cells, cell proliferation, cell scratch repair, and Transwell assays were conducted in A549 cells as shown in Figure 3. *CTSK*-OE significantly promoted the proliferation of A549 cells from the time point 48 onward (Figure 3a), significantly promoting cell migration and invasion (Figure 3b,c). In contrast, *CTSK*-KD inhibited the proliferation of A549 cells from the time point 72 onward (Figure 3a), significantly inhibiting cell migration and invasion (Figure 3b,c).

### 2.4. CTSK-OE Promoted While CTSK-KD Inhibited the Activation of the mTOR Signaling in A549 Cells.

**Figure 4:**
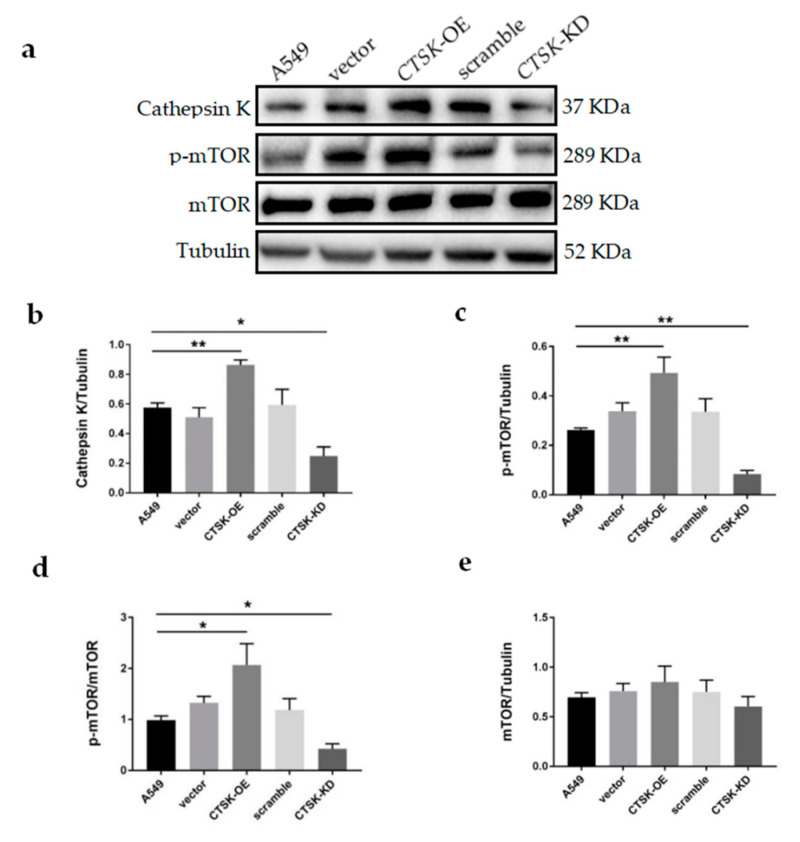
WB analysis for mTOR signaling protein expression in A549 cells. (**a**) Representative gel blots of Cathepsin K, p-mTOR, mTOR and Tubulin using specific antibodies. (**b**) Cathepsin K/Tubulin; (**c**) p-mTOR/Tubulin; (**d**) p-mTOR/mTOR; (**e**) mTOR/Tubulin. (mean ± SEM, *n* ≥ 3, * *p* ≤ 0.05, ** *p* ≤ 0.01, *** *p* ≤ 0.001).

In order to understand the mechanism of Cathepsin K in NSCLC, we detected mTOR and p-mTOR expression. The results are shown in Figure 4a. With a change in Cathepsin K expression levels (Figure 4b), *CTSK-*OE significantly increased level of p-mTOR and p-mTOR/mTOR, which promoted over-activation of the mTOR signaling pathway (Figure 4c,d). In contrast, *CTSK*-KD significantly decreased levels of p-mTOR and p-mTOR/mTOR, which inhibited activation of the mTOR signaling pathway (Figure 4c,d). No significant differences were found in the mTOR/Tubulin ratio among the groups (Figure 4e).

## 3. Discussion

In addition to normal physiological functions [26,27,28], Cathepsin K also exhibits deleterious effects on the body, as evidenced by its role in the progression of a variety of tumors. Over the past few years, accumulated data have shown overexpression of Cathepsin K in multiple cancer types, indicating its role in tumor progression and its potential diagnostic and prognostic values. For example, strong expression of Cathepsin K has been observed in primary melanoma and melanoma metastases [29]. Colorectal cancer is associated with high LPS secretion and overexpression of Cathepsin K [30]. In order to study the expression of Cathepsin K in NSCLC, human NSCLC cell lines A549 were selected in this experiment, and human embryo lung fibroblast MRC-5 cells were used as controls. WB and IF assays showed that the expression level of Cathepsin K in A549 was higher than that in MRC-5, which is consistent with the conclusion that Cathepsin K is highly expressed in cancer tissues and cells in the literature. Compared with MRC-5 cells in which Cathepsin K was slightly expressed in cells, Cathepsin K was largely expressed in A549 cells and diffused into the cytoplasm. Cathepsin K is isolated into lysosomes through the endosome, and it can also be secreted into the other compartments of the cell and extracellular environment [31,32], which is essential for its role in promoting the development of cancer cells in tumors. It is worth noting that Cathepsin K seems to also be distributed in the nucleus of MRC-5 cells. In general, the pathway for Cathepsin proteins entering the nucleus without nuclear localization sequence (NLS) has been a controversial issue. A recent study demonstrated that Cathepsin proteins could be “chaperoned” into the nucleus from a cytoplasmic source following possible leakages from the lysosome [33]. The mechanism of Cathepsin positioning to the nucleus and its role in the nucleus are also matters for further exploration.

Proteases play important roles in cancer initiation, development and metastasis. Cathepsin K–shRNA transfection has been demonstrated to downregulate Cathepsin K, inhibiting the proliferation and metastasis of breast cancer cells [34]. In squamous cell carcinoma, mesenchymal fibroblasts expressing Cathepsin K are secreted by tumor cells through interleukin-1 stimulation and are associated with tumor aggressiveness [35]. In vitro, Cathepsin K knockdown has been shown to inhibit migration and invasiveness of the OV-2008 cell line in epithelial ovarian cancer [36]. In order to explore the effects of Cathepsin K on the proliferation, migration, and invasion of NSCLC, this experiment used a transfection method to introduce a Cathepsin K recombinant plasmid and *CTSK*-siRNA into A549 cells to construct *CTSK*-OE and *CTSK*-KD experimental models. This research employed a two-way crossover study in order to investigate the role of Cathepsin K in A549 cells. In CCK-8 detection at 48 h, the results showed that cell proliferation in the *CTSK*-OE was significantly increased. At 72 h, *CTSK*-KD significantly decreased in the cell proliferation. From the time course prospective, it is likely because siRNA displays characteristics that are distinct from the recombinant plasmid [37,38], it plays a role at a longer time point in the experiment compared to the recombinant plasmid. This phenomenon was not observed in cell migration and invasion experiments, which might be due to the researchers’ selection of a specific time point for cellular detection.

In the cell scratch repair experiment, considering the damage of the transfection reagent to the cells, 1% fetal bovine serum was added to the culture medium. The cell proliferation cycle is generally 24 h in length. In order to exclude the effect of serum on migration experiments, the time point of 24 h was selected for the detection. In the same manner that the invasion experiment was conducted, no serum was present in the upper chamber of Transwell, but the medium in the lower chamber contained a serum concentration of 20%. The large concentration gradient between the upper and lower chambers resulted in a tendency for the A549 cells to be preferentially located in the lower chamber. The time point at 24 h was selected in order to avoid the potential proliferation of cells after penetration of the polycarbonate membrane, a factor that could influence the accuracy of the experiment. The results of the migration and invasion experiments were consistent with the results of the cell proliferation experiments. *CTSK*-OE significantly enhanced the migration and invasion ability of A549 cells. In contrast, the migration and invasion ability of *CTSK*-KD was significantly reduced. This is consistent with the conclusion that Cathepsin K is highly expressed during tumor invasion and metastasis [39] and has a stimulating effect on the aggressive phenotype of various types of cancer. Soond et al. [40] reported that the findings of use of utilizes combined chemotherapeutic treatment such as with Tocilizumab or Rituximab inhibited signaling transduction pathways which up-regulate the intracellular Cathepsins look very encouraging for targeting cancer. In the gastric metastasis model, the use of the pharmaceutical agent Odanacatib significantly inhibited the metastases of cancer cells, suggesting that Cathepsin K inhibition may be employed as a new therapeutic strategy to prevent tumor metastasis [41,42,43].

**Figure 5:**
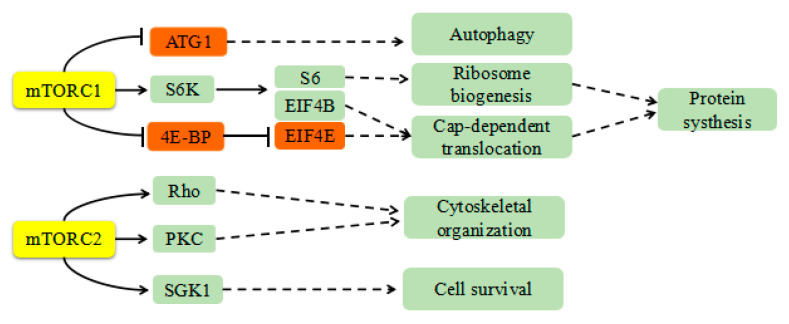
The role of mTOR in vivo. mTOR promotes protein synthesis by phosphorylating its substrates S6 and EIF4E1. It can further activate downstream products of eIF4 complex to promote tumor development, regulate the cell cycle, and inhibit autophagy and apoptosis.

In addition to its well-known role in extracellular matrix degradation and remodeling, Cathepsin K may mediate activation of the mTOR signaling pathway. mTOR promotes anabolism and protein synthesis by phosphorylating its substrates S6 and EIF4E1 [44,45,46], as shown in Figure 5. It can further activate downstream products of the eIF4 complex to promote tumor development, regulate the cell cycle, and inhibit autophagy and apoptosis [19]. Seo et al. [25] demonstrated that inhibition of mTOR enhanced the chemosensitivity of cancer cells. They also revealed that treatment with mTOR inhibitors reduced tumor size and increased apoptosis in a xenograft model. Evimus, an inhibitor of mTOR, selectively inhibits mTOR signaling. It has been comprehensively evaluated in several phase I trials of previously treated advanced NSCLC [47].

In this experiment, *CTSK-*OE significantly increased levels of p-mTOR and p-mTOR/mTOR, which promoted over-activation of the mTOR signaling pathway. In contrast, *CTSK*-KD significantly decreased levels of p-mTOR and p-mTOR/mTOR, which inhibited activation of the mTOR signaling pathway. Previous studies have demonstrated that inhibition of Cathepsin K can induce proteasomal degradation of proteins associated with regulatory mechanisms on the target of rapamycin [25]. This conclusion is inconsistent with our experiment, which suggests that Cathepsin K may play a role in NSCLC through regulation of the mTOR signaling pathway. However, the mechanism of action by which Cathepsin K promotes the proliferation, migration, invasion, and other malignant behavior of NSCLC cells is unclear. Potential mediators involved in the effects of Cathepsin K could include a certain molecule’s unique and powerful hydrolytic activity or its role in the mTOR signaling pathway. In addition, whether the changes of Cathepsin K in NSCLC cells are related to the levels of p-mTOR remains unclear. Therefore, it is necessary to supplement the Cathepsin K activity detection and mTOR signaling pathway inhibitor experiments in further research.

In summary, our results demonstrated that Cathepsin K was overexpressed in NSCLC cells and permeated the cytoplasm. Through the detection of cell biological functions, it was found that increased expression of Cathepsin K promoted the proliferation, migration, invasion, and activation of the mTOR signaling pathway of NSCLC cells, while silencing Cathepsin K expression reversed the above behavior. These findings suggest that Cathepsin K may potentially represent a new therapeutic target for NSCLC.

## 4. Materials and Methods 

### 4.1. Cell Culture and Transfection

The MRC-5 and A549 cells were maintained in Dulbecco’s modified Eagle’s medium (DMEM) (Sangon Biotech, Shanghai, China) supplemented with 10% fetal bovine serum (FBS) (ExCell Bio, Beijing, China) and cultured in 5% CO_2_ at 37 °C. The A549 cells were stably upregulated and downregulated for Cathepsin K expression using the Cathepsin K plasmid and siRNA (Genepharma, Shanghai, China), respectively. DNA transfection was performed using Lipofectamine-3000 (Thermo Fisher, Waltham, MA, USA), and siRNA was transfected at a final concentration of 50 nM using Lipofectamin-3000.

### 4.2. qRT-PCR

Total RNA was isolated using RNAiso Plus (TaKaRa, Kusatsu-shi, Japan), and RNAs were quantified using a NanoDropTM 2000 spectrophotometer (Thermo Fisher Scientific, Waltham, MA, USA). Synthesis of cDNA and reverse transcription was performed using 1 μg of total RNA in a 25 μL system following the instructions of a FastQuant RT Kit (Tiangen, Beijing, China). *CTSK* and *GAPDH* primers for qPCR were designed by PrimerPremier5 software. The sequences were *CTSK*-F: 5’-CCTTGAGGCTTCTCTTGG-3’ *CTSK*-R: 5’-AGGGTGTCATTACTGCGG-3’; *GAPDH-F*: 5’-AGAAGGCTGGGGCTCATTTG-3’ *GAPDH-F*: 5’-AGGGGCCATCCACAGTCTTC-3’. Quantitative real-time PCR was performed for *CTSK* and *GAPDH* (housekeeping gene) using a C1000 Touch Thermal Cycler CFX96TM Real-Time System (Bio-Rad, Shanghai, China) per the Universal SYBR Green qPCR Supermix (UE, Suzhou, China) instructions. Real-time PCR was triplicated for each cDNA sample.

### 4.3. Western Blot Analysis

Total protein was isolated using radio immunoprecipitation assay (RIPA) lysate (strong) (Solarbio, Beijing, China), and the whole process was performed on ice. Proteins were quantified using a Microplate reader (Molecular Devices, Silicon Valley, CA, USA) following the instructions of a bicinchoninic acid (BCA) protein quantification kit (Solarbio, Beijing, China). Protein samples were then separated onto SDS-polyacrylamide gels and transferred electrophoretically to polyvinylidene fluoride (PVDF) membranes. The membranes were blocked with 5% milk and incubated overnight at 4 °C with anti-CathepsinK (1:1000; ab19027), anti-mTOR (1:1000; CST2983), anti-p-mTOR (1:1000; CST5536), anti-GAPDH (1:10,000; Proteintech10494-1-p), and anti-Tubulin (1:1000; CST2144). Blots were incubated with a horseradish peroxidase (HRP)-conjugated secondary antibody (1:3000; CST7074). Antigens were detected using a luminescence method. Band densities were determined using Image Lab software (version 5.1, Bio-Rad).

### 4.4. Immunofluorescence Assays

The cells were incubated successively for fixation and permeation with 4% paraformaldehyde (Leagene Biotechnology, Anhui, China) and 0.1% TritonX-100 (Leagene Biotechnology, Anhui, China). After successively incubating the cells with the configured PBST (phosphate buffer saline (PBS) (Sangon Biotech, Shanghai, China) + 0.1% Tween 20 + 1% BSA + 22.52 mg/mL Glycine) to block the non-specific binding of the antibody, the slides were rinsed and incubated with the appropriate primary antibody for Cathepsin K (1:100) overnight at 4 °C. The following day, the slides used for IF staining were incubated with fluorescein-conjugated goat anti-rabbit IgG and subsequently stained with nuclear dye 4′,6-diamidino-2-phenylindole (DAPI). Finally, the slides were then examined and imaged using an Olympus fluorescence microscope (fv3000), and the fluorescence intensity was calculated using Image J software (version 1.48v). In this experiment, 3 images were collected in each group, and each image outlines 10 cells for fluorescence intensity analysis.

### 4.5. Proliferation Assays

A Cell Counting Kit (APE×BIO, Houston, TX, USA) was used to evaluate the variation in cell proliferation, which is based on the dehydrogenase activity detected in viable cells. The formazan dye generated by dehydrogenases absorbs light at a wavelength of 450 nm. The amount of formazan dye present in cells is directly proportional to the number of living cells. In brief, 100 μL of cell suspension (1 × 10^3^) was incubated in 96-well culture plates, and 10 μL of CCK-8 solution was added at the time set by the experiment. Cells were incubated at 37 °C for 2 h. Absorbance was analyzed at 450 nm using a microplate reader.

### 4.6. Migration Assays

A migration assay was used to detect the variation in cell migration. A549 cells were plated in 6-well plates at a certain amount (1 × 10^6^) and allowed to form a confluent monolayer for 24 h. The monolayer was scratched with a sterile pipette tip (10 μL), followed by a wash with PBS to remove floating and detached cells. The cells were then imaged (at time 0 h and 24 h) by fluorescent inversion fluorescence microscopy (Olympus, Tokyo, Japan). Image J software was used to measure the scratch area and length of each image at 0 h and 24 h. According to the formula area/length, calculate the scratch width of the two periods and make the difference of the corresponding image, and the scratch width difference/corresponding initial width is the respective migration of each group.

### 4.7. Invasion Assays

The invasion assay was performed using a Transwell 24-well dish with a pore size of 8 μm (Costar, NY, USA). Mix the serum-free DMEM medium and Matrigel (Corning, Corning, NY, USA) at a ratio of 6:1 on ice, and added 60 μL to the Transwell chamber. 100 μL of DMEM serum-free medium cell suspension (5 × 10^4^) was placed in the upper chamber, and 500 μL of DMEM medium containing 20% serum was placed in the lower chamber. The cells were incubated for 24 h at 37 °C in 5% CO_2_, then fixed in 4% methanol and stained with 0.1% crystal violet. Cells on the upper side of the filters were removed with cotton-tipped swabs and the filters were then washed with PBS. Cells on the underside of the filters were examined and counted under a microscope.

### 4.8. Statistical Analysis

Results were representative of at least three independent experiments, and all values were expressed as mean ± SEM. Statistical significance (*p* < 0.05) for each variable was estimated by an unpaired *t*-test (two-tailed) or a one-way analysis of variance (ANOVA) followed by a Tukey’s post hoc analysis.
